# Challenges and Innovations in Paediatric Brain Tumour Treatments

**DOI:** 10.3390/pharmaceutics17121561

**Published:** 2025-12-03

**Authors:** Nadia Shah, Jessica Sohl, Massimo Ganassi, Loredana Guglielmi, Sara Badodi

**Affiliations:** 1 Blizard Institute, Barts and The London School of Medicine and Dentistry, Queen Mary University of London, London E1 2AT, UK; 2 Genetic Therapy Accelerator Centre, Department of Neurodegenerative Diseases, University College London, London WC1N 3BG, UK; m.ganassi@ucl.ac.uk; 3 Faculty of Health and Medical Sciences, School of Biosciences, University of Surrey, Guildford GU2 7HX, UK

**Keywords:** tumour, brain, paediatric, drug, immunotherapy, treatment, epigenetic, signalling pathways

## Abstract

Paediatric brain tumours are the most common solid tumours and the leading cause of cancer-related mortality in children. Current treatment, which includes surgery, chemotherapy, and radiotherapy, has improved survival for some types of them but is associated with significant long-term side effects that reduce the quality of life. These unique limitations highlight the urgent need for novel therapeutic strategies tailored to the specific biology and vulnerabilities of the developing brain. This review focuses on clinical trials conducted over the past decade in paediatric brain tumours to elucidate emerging therapeutic directions. By analysing trial phases, type of interventions, and molecular targets, we aimed to identify trends that could inform future treatment development. Our findings show that targeted therapies, particularly kinase inhibitors, epigenetic modulators, and immunotherapies, represent the most frequently explored interventions, although most trials remain in early clinical phases. Diagnostic innovations, including hybrid PET/MRI and molecular profiling, highlight a shift to precision medicine. Collectively, these trends reveal a diversifying therapeutic field that may, with international collaboration and long-term safety evaluation, transform outcomes for children with paediatric brain tumours.

## 1. Introduction

Cancer is a major cause of mortality in children and adolescents across the world. Every year, an estimated 400,000 children and adolescents aged 0–19 years old develop cancer worldwide [1]. Paediatric brain tumours (pBTs) are the most common solid tumours, accounting for 25% of all paediatric cancers, which represent the leading cause of tumour-related illness and death in childhood [2,3,4].

pBTs represent a diverse group of diseases, with different sites of formation and clinical behaviour [5] (Table 1). The most common types of high-grade BT in children include medulloblastoma (MB), ependymoma (EPN), diffuse midline glioma (DMG), high-grade glioma (HGG), Atypical Teratoid/Rhabdoid Tumour, and Embryonal Tumour with Multilayered Rosettes (Table 1).

**Table 1:** Key clinical features of major paediatric brain tumour types. Summary of common aggressive paediatric brain tumours, including their typical anatomical location, prognosis, age distribution, and relative frequency.

Tumour Type	Location	Prognosis	Age Group	% of Cases
**Diffuse Midline Glioma [6,7]**	Midline locations	Very Poor (<10% survival)	6–10 years	~10–20%
**High-Grade Glioma [7,8]**	Cerebral hemispheres	Poor (<20% survival)	Adolescents >10 years	~8–12%
**Medulloblastoma [9]**	Cerebellum	Variable (~50–70% for high-risk)	Peak: 5–9 years	~20%
**Atypical Teratoid/Rhabdoid Tumour [10]**	Mostly cerebellum	Poor (~30–50% survival)	Infants and young children (median ~3 years)	~2%
**Ependymoma [11]**	Mostly in fourth ventricle	Poor (~50% survival)	Median: ~4–5 years	~10%
**Embryonal Tumour with Multilayered Rosettes [12]**	Variable	Poor (<30% survival)	Infants and young children	~2%

The treatment of pBTs frequently includes tumour resection followed by chemotherapy and radiation therapy; however, despite ongoing research, there is still a high unmet need for novel therapies that can improve survival outcomes and quality of life for patients [13]. Outcomes remain poor for many high-grade pBTs with long-term effects on survivors due to the toxicity of treatments, particularly chemotherapy and radiotherapy, which are associated with significant neurocognitive side effects. In particular, pBTs present specific therapeutic challenges that are distinct from those encountered in adults. The developing brain is exposed to the long-term toxicity of conventional therapies, resulting in neurocognitive impairment, endocrine dysfunction, and impaired psychosocial development. Hence, treatment in children must balance efficacy with maintenance of the quality of life over decades of survivorship. Biologically, paediatric tumours exhibit different genetic and epigenetic landscapes compared to their adult counterparts, often characterised by fewer mutations but greater dysregulation of developmental signalling pathways, requiring the development of paediatric-specific treatment strategies.

Despite advances in surgery, chemotherapy, and radiotherapy, the management of pBTs remains challenging due to several biological and clinical limitations. A major challenge to effective treatment of pBTs is the blood-brain barrier (BBB), which tightly regulates molecule penetration into the central nervous system (CNS) and prevents many drugs from reaching the tumour site at therapeutic concentrations [14]. Additionally, pBTs are characterised by intra- and intertumoral heterogeneity, with distinct cellular subpopulations contributing to therapy resistance and recurrence [15,16]. Furthermore, the location of these tumours further complicates surgical resection, and even successful surgery may result in long-term neurocognitive and developmental consequences [17,18]. Together, these factors underscore the urgent need for novel and more precise therapeutic strategies to overcome these barriers and improve both survival and quality of life of the affected children.

Here, we review the recent advancements in treatment strategies and clinical trials over the past decade, with the aim of identifying trends in therapeutic approaches, including immunotherapy, targeted therapy, and combination treatments.

## 2. Trends in Paediatric Brain Tumour Clinical Trials over the Past Decade

**Figure 1:**
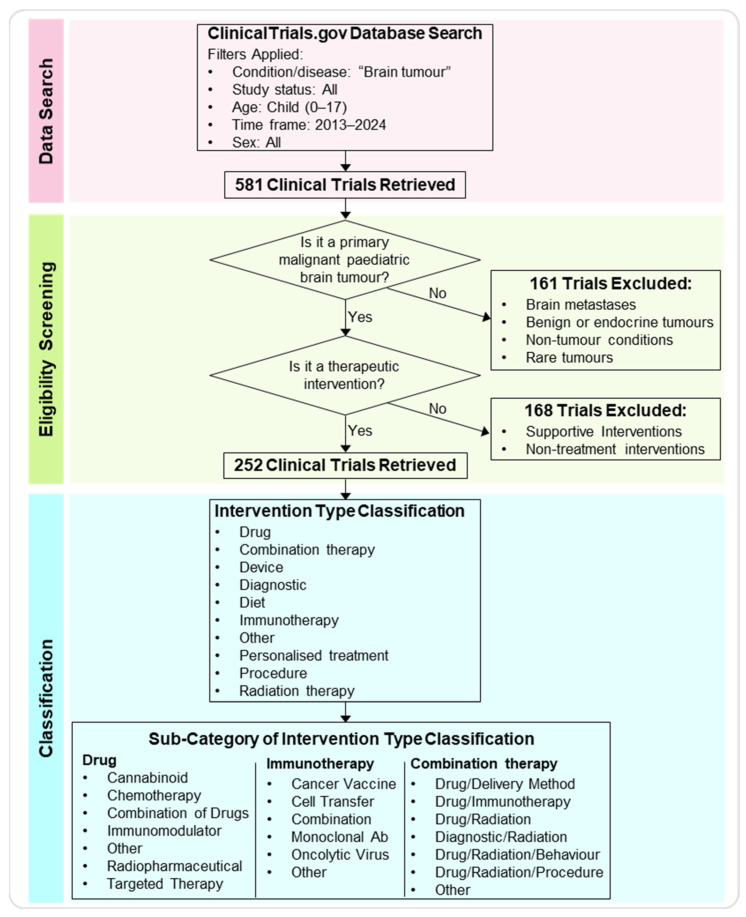
Workflow of the data search, eligibility screening, and classification analysis. Workflow diagram depicting the main steps of the meta-analysis. The ClinicalTrials.gov database was searched for paediatric brain tumour trials between 2013 and 2024 (ages 0–17). After applying inclusion and exclusion criteria, 252 trials were selected for analysis. These were categorised by intervention type categories and sub-categories.

**Figure 2:**
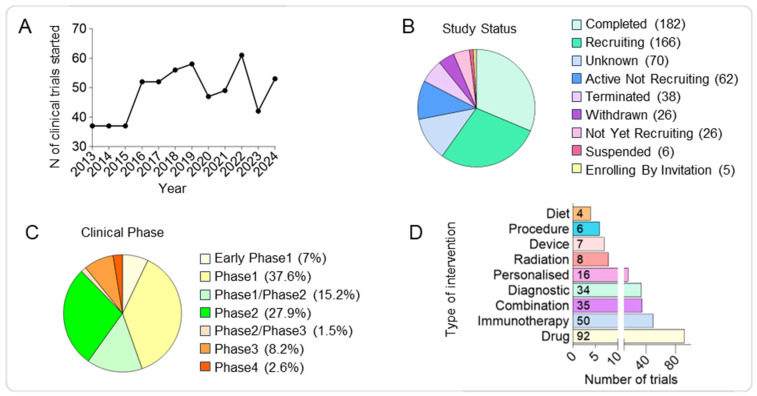
Competitive landscape of clinical trials in paediatric brain tumours over the past decade. (**A**) Line graph showing the annual number of clinical trials initiated between 2013 and 2024, demonstrating a generally steady output with year-to-year variability. (**B**) Pie chart showing the distribution of clinical trials by recruitment status, including recruiting, active but not recruiting, completed, terminated, withdrawn, and suspended studies. (**C**) Pie chart showing the distribution of trials by clinical phase, highlighting the predominance of early-stage studies. (**D**) Histograms showing the number of trials by intervention type, with drugs comprising the largest category, followed by immunotherapy, combinations, and diagnostics, while dietary, device, procedure, and radiation interventions represent a smaller fraction.

We searched the U.S. National Library of Medicine Clinical Trials (ClinicalTrials.gov) online database to identify clinical trials on pBT treatments for patients aged 0–17 (tagged as ‘child’) in the past 10 years, including ongoing, completed, and terminated trials (Figure 1 and Figure 2A,B).

For such analysis, 581 clinical trials were obtained from the filtered database search (Supplementary Table S1). Among the trials with available information about the phase of the study, we observed most of them to be in Phase1 (38%), an initial stage required to assess safety, tolerability and optimal dosage, followed by Phase2 trials (28%) focussed on evaluating the efficacy and to further assess the safety at the optimal dose and Phase1/Phase2 (15%) representing combined trials to streamline early safety and efficacy assessments. Around 10% of the analysed trials are currently in the most advanced study phases (8.2% in Phase3 and 2.6% in Phase4) required to confirm efficacy, monitor side effects, and compare to standard treatments and finally monitor long-term safety and effectiveness; while 7% of the trials are in the Early Phase1 representing exploratory studies with limited human exposure to determine pharmacokinetic (PK) and pharmacodynamics (PD) profiles. Finally, the lower percentage of the analysed study phase is represented by Phase2/Phase3 trials, combined trials to confirm the efficacy and gather more safety data before proceeding to Phase3 (Figure 2C and Supplementary Table S2).

First, we further refined the obtained clinical trials database by ensuring that (a) the trial focused on more prevalent primary malignant pBTs and (b) verifying that the intervention proposed in the trial was a treatment. Brain metastases, endocrine brain tumours, rare brain tumours, and other brain conditions such as Sturge-Weber Syndrome and Intracranial Arteriovenous Malformations were excluded, as were treatments involving supportive intervention for primary analysis and non-treatment interventions (e.g., neurocognitive testing, rehabilitation programs, and quality of life questionnaires). After screening and assessing eligibility, 252 trials were analysed, while 329 trials were excluded (161 were excluded because the trial did not include primary malignant brain tumours, and 168 were excluded because the proposed intervention was not a treatment).

Second, the intervention applied in each clinical trial was analysed and sorted into ten defined categories: drug, immunotherapy, combination therapy, diagnostic procedure, personalised treatment, radiation therapy, procedure, diet, device, or other. If a study proposed an intervention in addition to the gold standard of care for a brain tumour (surgery, chemotherapy, and radiation) without labelling it as a combination study, the intervention was categorised independently rather than as combination therapy. Of the analysed clinical trials, the most common intervention involved the use of a drug (37%), followed by immunotherapy (20%), combination therapy (14%), diagnostic (13%), personalised (6%), radiation (3%), device (3%), procedure (2%), and diet (1%) (Figure 2D).

This meta-analysis highlights the rapid diversification of therapeutic modalities that are currently explored for pBTs. In the following sections, we discuss how these trends translate into specific targeted, epigenetic, and immune-based therapeutic strategies. While multiple other treatment modalities, such as metabolic therapies, radiopharmaceuticals, and novel delivery systems, are emerging, this review focuses on the most represented intervention types identified in our analysis to provide a detailed overview of their current clinical development.

## 3. Precision Targeted Approaches in Paediatric Brain Tumours

### 3.1. Kinase Inhibitors as Leading Targeted Therapies for Paediatric Brain Tumours

**Figure 3:**
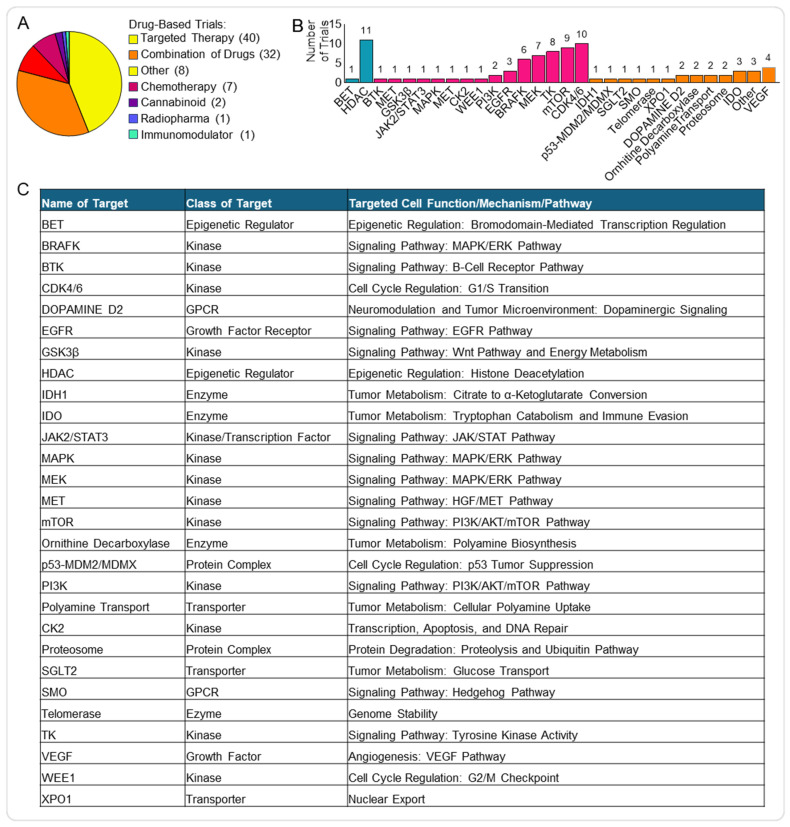
Targeted therapies in paediatric brain tumour clinical trials. (**A**) Pie chart showing the distribution of drug-related clinical trials. (**B**) Histogram displaying the frequency of specific molecular targets among targeted therapy trials, including epigenetic factors (blue) and kinases (pink). (**C**) Table summarising major molecular targets, their functional class, the associated cellular role, and the key pathways affected. List of abbreviations in Supplementary Table S3.

We defined a drug as any medication or substance designed to target cancer cells or disrupt the processes involved in cancer growth, progression, or metastasis. Further, we have classified the drug’s clinical trials into additional categories to better clarify the type of substance used, identifying targeted therapy, combination of drugs, chemotherapy, cannabis, radiopharmaceutical, immunomodulatory and other drugs (Figure 3A). Drugs used in targeted therapy, inhibiting specific genetic or molecular pathways involved in the tumour biology, represented the most common drug treatment (40/92, 43%), followed by a combination of drugs (32/92, 35%) (Figure 3A).

We combined the targeted therapies used as single and combined treatments (Figure 3B,C) and found that the most common type was kinase inhibitors (50/86, 58%). Kinase inhibitors block specific enzymes (i.e., kinases) responsible for phosphorylation events involved in multiple signalling pathways frequently deregulated in cancer and essential for tumour cell proliferation, migration and invasion, but also for response and resistance to therapy [19].

Among the targeted therapy clinical trials, the most prevalent kinase inhibitors were cyclin-dependent kinase 4/6 inhibitors (CDK4/6i) (10/50, 20%) and mechanistic target of rapamycin inhibitors (mTORi) (8/50, 16%) (Figure 3B).

### 3.2. Key Molecular Pathways Targeted in Paediatric Brain Tumours

**Figure 4:**
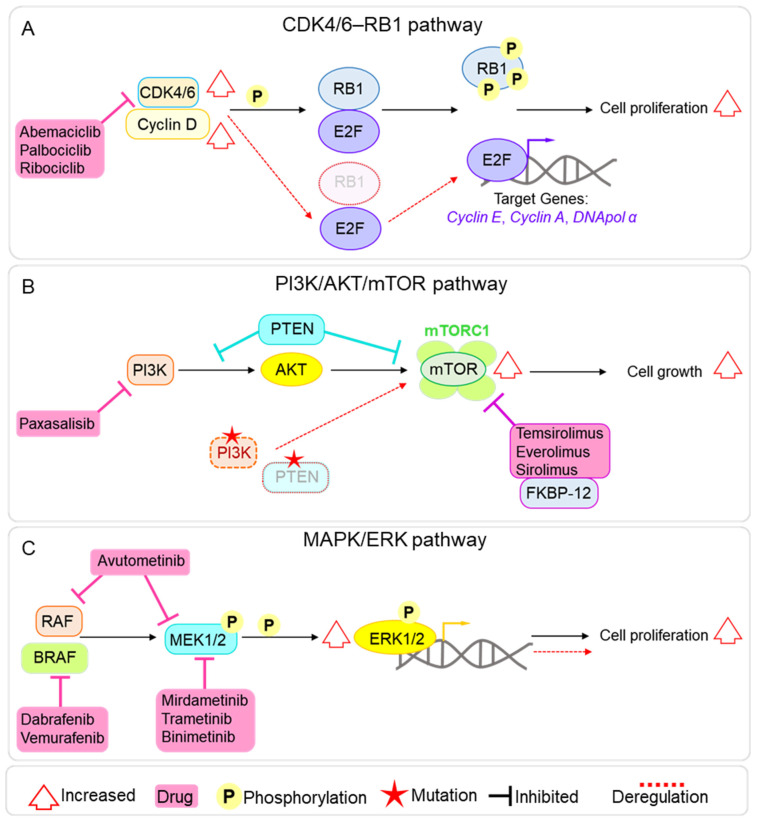
Main molecular pathways targeted by clinical trials in paediatric brain tumours. (**A**) Diagram of the CDK4/6–RB pathway, showing its role in cell cycle progression, common mechanisms of deregulation in paediatric brain tumours (in red), and the points of inhibition by the CDK4/6 inhibitors Abemaciclib, Palbociclib, and Ribociclib. (**B**) Diagram of the PI3K/AKT/mTOR pathway, illustrating upstream and downstream signalling components, frequent alterations observed in paediatric brain tumours (in red), and the sites of action of main mTOR inhibitors used in analysed clinical trials. (**C**) Diagram of the MAPK/ERK signalling cascade, highlighting activation via growth factor receptors, recurrent aberrations in paediatric brain tumours (in red), and the inhibition points targeted by MEK and BRAF inhibitors.

The CDK4 and CDK6 kinases have similar structure and function [20] and regulate the cell cycle progression through the G1/S checkpoint. CDK4/6, together with Cyclin D, forms an active complex that phosphorylates the retinoblastoma protein 1 (RB1), leading to the release of the E2F transcription factor. E2F then activates the transcription of key genes (e.g., Cyclin E, Cyclin A, and DNA polymerase α) essential for G1/S transition and DNA replication (Figure 4A) [21]. Dysregulation of the CDK4/6 pathway leads to increased cell proliferation, making inhibition of these kinases a promising approach to cancer treatment [22], with some CDK4/6 inhibitors currently approved for the treatment of breast cancer [23]. Interestingly, the CDK4/6 axis is also dysregulated in pBTs. This can occur mainly through genetic alterations, including loss of *RB1* [24] and *CDKN2A* [25], and amplification of *CCND1* (Cyclin D1) [26] or *CDK4* [24,27] (Figure 4A). Abemaciclib, Palbociclib, and Ribociclib are the CDK4/6 inhibitors that we found to be currently trialled alone or in combination (mainly with chemotherapy and mTOR inhibitors) for pBTs and demonstrated efficacy in both pre-clinical and clinical settings [28,29]. Interestingly, these drugs are already approved for the treatment of breast cancer. These small-molecule inhibitors bind to the ATP-binding pocket on CDK4/6, preventing the kinase activity of the CDK4/6-Cyclin D complex and, in turn, blocking RB1 phosphorylation and inducing a G1 arrest in the cell cycle.

mTOR is a serine/threonine kinase that plays an essential role in the regulation of several cellular functions, including cell growth, proliferation, division, and metabolism. mTOR forms two distinct complexes with different components and regulation, which mediate distinct downstream effects. mTORC1 controls metabolism and cell growth, and mTORC2 modulates cell proliferation and survival [30]. The mTOR signalling pathway is commonly overactive in cancer, leading to uncontrolled cell proliferation and metabolic rewiring, making it an attractive target for treatment [31,32]. mTOR deregulation is also found in pBTs, including pHGG, MB, and EPN, and can be mediated by mutations in upstream regulators (e.g., *PIK3CA* and *PTEN*) or loss of PTEN function, which negatively regulates the PI3K/AKT/mTOR axis [33] and constitutive mTOR activation [34] (Figure 4B). mTOR inhibitors are currently approved to treat various cancers, such as renal cell carcinomas, neuroendocrine tumours, and breast cancer [35] and have shown promise as a treatment option for pBTs, both in pre-clinical and clinical settings [36,37,38,39]. Four main mTOR inhibitors are currently under clinical trial for pBTs: Temsirolimus, Everolimus, and Sirolimus inhibit mTORC1 by forming a complex with FKBP12, which binds to the FRB domain of mTOR and allosterically suppresses mTORC1 kinase activity; the fourth is the dual PI3K/mTOR inhibitor Paxalisib (GDC-0084), specifically designed to cross the BBB [40] (Figure 4B).

Finally, we found several clinical trials targeting kinases involved in MAPK/ERK signalling: BRAF (6/50), MEK (6/50), and MAPK (1/50). The MAPK/ERK pathway plays a crucial role in many cellular processes, including cell proliferation, differentiation, survival, and stress response [41], and its deregulation can lead to tumour formation and progression and can be involved in therapy resistance [42]. MAPK/ERK overactivation has been reported in several pBTs, including MB [43,44], EPN [45], and glioma [46]; hence, targeting the pathway represents an attractive avenue for therapeutic approaches. In normal cells, the MAPK signalling is activated by extracellular signals via Receptor Tyrosine Kinases on the cell surface and the activation of RAS GTPase, which in turn activates a cascade of phosphorylation events on RAF kinases, including BRAFK (B-Raf kinase), MEK (Mitogen-Activated Protein Kinase Kinase), and MAPK (Mitogen-Activated Protein Kinase, also known as ERK) (Figure 4C). Finally, the activated MAPK/ERK kinase translocates to the nucleus, where it phosphorylates and activates several transcription factors. Among the analysed targeted therapies’ clinical trials, we found several small molecules targeting the whole axis of the signalling pathway (Figure 4C): Mirdametinib, Trametinib, and Binimetinib (MEK162), which target MEK1/2; Dabrafenib and Vemurafenib, which inhibit BRAFK; and the dual RAF/MEK inhibitor Avutometinib.

### 3.3. Epigenetic Modulators in Paediatric Brain Tumour Treatment

The second most common targeted therapy is represented by inhibitors of epigenetic factors (11/86, 13%), with histone deacetylase inhibitors (HDACi) making up most of this group (10/11, 91%).

Epigenetic regulation is a fundamental mechanism that governs gene expression without altering the underlying DNA sequence. Key epigenetic events include DNA methylation, histone modification (e.g., acetylation, methylation, phosphorylation), and chromatin remodelling, which together modulate the accessibility of transcriptional machinery to the genome. These mechanisms have tight spatial and temporal control to ensure proper development, differentiation, and cellular homeostasis [47].

Epigenetic dysregulation plays a crucial role in pBTs, where mutation frequency is low [48,49] and contributes to aberrant gene expression and cellular transformation. Global DNA hypomethylation and hypermethylation at the promoter of tumour suppressor genes, histone mutations disrupting histone post-translational modifications, and deregulation of epigenetic factors (e.g., HDACs and DNA methyltransferases) have been reported in various pBTs [50] highlighting the potential of developing therapeutic approaches targeting epigenetic regulation. HDAC activity is indeed deregulated in pBTs and is involved in tumour formation and growth; for example, HDAC5 and HDAC9 are highly expressed in MB and are associated with poor overall survival [51].

The main HDAC inhibitors used in the analysed clinical trials are Panobinostat, Vorinostat, Valproic Acid, HBI-8000, Entinostat, MTX110, and Fimepinostat. All these inhibitors primarily target Class I HDACs (HDAC1, HDAC2, HDAC3), with some (e.g., Panobinostat, Vorinostat, HBI-8000) also targeting Class IIb HDAC6, which regulates non-histone proteins like α-Tubulin. Interestingly, Fimepinostat can also inhibit the PI3K pathway.

Targeted therapies represent a promising approach for treating pBTs, with preclinical and early clinical data supporting their efficacy. However, their clinical translation might be limited by tumour heterogeneity, BBB penetration, and developmental toxicity, as many pathways targeted are essential for normal brain development. Single-agent therapy often fails due to adaptive resistance and compensatory signalling, highlighting the need for rational combination strategies. Moving forward, the success of targeted therapy in pBTs would depend on robust molecular profiling, careful patient selection, and combinatorial approaches that balance efficacy with long-term safety.

## 4. Immunotherapy Approaches Used for the Treatment of Paediatric Brain Tumours

Beyond molecularly targeted and epigenetic interventions, immunotherapy has emerged as another approach in paediatric neuro-oncology. We defined immunotherapy as any treatment that harnesses, stimulates, and/or modifies the body’s immune system to fight cancer. There are several immunological limitations which make pBTs challenging to treat, with a major one being the tumour microenvironment (TME) [52]. The TME differs between tumours and presents several immunosuppressive factors. This includes the secretion of immunosuppressive cytokines such as TGF-β, IL-10, and IL-6. The blocking of TGF-β signalling leads to an increase in CD8-T cell-mediated cytotoxicity due to a reduction in regulatory T cells. The secretion of IL-6 leads to the activation of the JAK/STAT signalling pathway, which is responsible for several immunosuppressive functions [53]. Additionally, within the TME are tumour-associated macrophages/microglia and myeloid-derived suppressor cells, all of which have immunosuppressive functions that reduce the effective killing of tumour cells. Additional challenges in pBTs include the heterogeneity of target antigen expression and their classification as immunologically “cold” due to the low T-cell infiltration [54].

**Figure 5:**
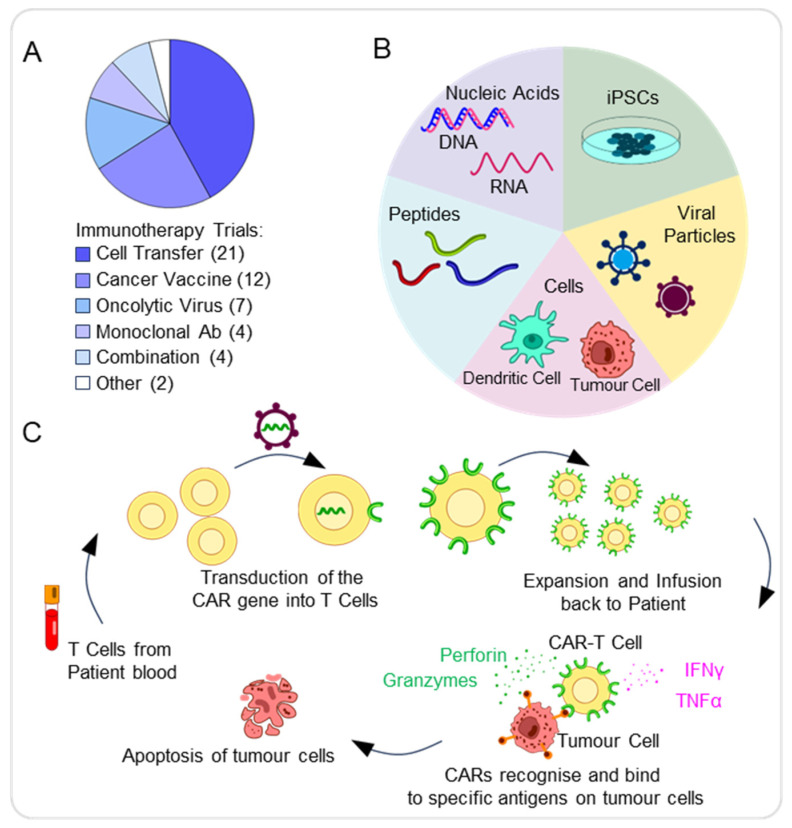
Immunotherapy approaches in paediatric brain tumours. (**A**) Pie chart showing the distribution of immunotherapy approaches for the treatment of pBTs. (**B**) Schematic summarising the various types of cancer vaccine approaches available, including cell, viral, peptide, iPSC, and nucleic acid-based vaccines. (**C**) Schematic representing the process to generate and activate chimeric antigen receptor (CAR)-T cells, involving collecting the patient’s blood and from these, leukocytes, including T cells, can be extracted, with transduction of CARs into T cells and expansion of CAR-T cells before infusion into patients. CAR-T cells bind to specific antigens on tumour cells via the antigen recognition domain, leading to their activation. As a result, cytokines, including IFN-γ or TNF-α, are released, as well as granzymes and perforin. The release of cytolytic molecules then leads to the destruction of tumour cells via apoptosis.

Altogether, these features make pBTs difficult to treat, and hence, the development of targeted immunotherapies is essential to overcoming these specific limitations. We found that the most common type of immunotherapy was cell transfer (21/50, 42%), followed by cancer vaccines (12/50, 24%) and oncolytic viruses (7/50, 14%). The most common type of cell transfer was chimeric antigen receptor (CAR) T-cell therapy (CAR-T) (10/21, 48%) (Figure 5A).

### 4.1. Cancer Vaccines

Cancer vaccines aim to stimulate immune responses against tumour-specific antigens. The different types of cancer vaccines include peptide, nucleic acid, cell (including tumour and dendritic cells), viral, and induced pluripotent stem cell (iPSC)-based vaccines (Figure 5B).

A multicenter trial of an H3.3K27M vaccine in diffuse intrinsic pontine glioma (DIPG) or DMG demonstrated tolerability and immunological responses. Patients showing a CD8^+^ T-cell response to the vaccine achieved a median overall survival of 16.3 months, in comparison to 9.9 months in non-responders [55].

Another Phase1 trial will begin to investigate SurVaxM in paediatric and young adult patients with progressive or relapsed MB, HGG, EPN, and newly diagnosed DIPG (NCT04978727). SurVaxM targets SURVIVIN, a protein highly expressed in tumour cells but not normal cells and thus should result in the specific inhibition of tumour growth.

In parallel, a Phase1 trial of CMV RNA-pulsed dendritic cells (DC) treatment is ongoing in children and young adults with grade IV glioma, recurrent malignant glioma, or recurrent MB. Early results are promising as no serious adverse events have been documented; however, further results are needed to assess the efficacy of this treatment (NCT03615404).

Additional cancer vaccines include the use of DCs pulsed with tumour-specific mRNA that has been produced from the patient’s tumour cDNA libraries [52]. A current DC vaccine being investigated consists of *WT1* mRNA-loaded autologous monocyte-derived DCs. This study included 10 paediatric patients with newly diagnosed HGG and DIPG to assess the feasibility and safety of the treatment, as well as patients’ quality of life (NCT04911621).

### 4.2. CAR-T Cell Therapy

Another form of immunotherapy is the use of chimeric antigen receptor (CAR-T) cells. Through genetic modifications, T-lymphocytes express synthetic receptors known as CARs, enabling them to target and eliminate cancer cells presenting specific tumour-cell antigens [13,56]. CARs consist of four key elements: the antigen-binding domain, hinge region, transmembrane domain, and at least one signalling domain. These allow for the CARs to achieve specificity and stable expression, thus resulting in an anti-tumour response (Figure 5C) [56]. The use of anti-CD19 CAR-T cells for the treatment of recurrent B-cell lymphoma in children has been promising, as CAR-T cells successfully destroy B cells that express the CD19 antigen [57]. Due to the successful use of CAR-T cells in other cancers, including paediatric, the use of CAR-T cells for the treatment of pBTs seems promising.

EGFR is known to be highly expressed in both adult and pBTs, including glioblastomas, EPNs, and MBs [58,59]. An in vivo study investigating EGFR806-CAR-T cells in NSG mice implanted with U87 glioma showed that these cells effectively killed glioma cells and eradicated tumours without recurrence, whilst leaving healthy foetal astrocytes unaffected [60]. A Phase1 clinical study is now underway to investigate the efficacy and safety of EGFR806 CAR-T cells for the treatment of recurrent or refractory EGFR-positive paediatric CNS tumours (NCT03638167).

Experimental data also suggest GD2 as a potential therapeutic target for pBTs, as it is highly expressed in Group 3, Group 4, and SHH MB [61]. In NSG mouse models with MB, treatment with GD2-CAR-T cells for 2 weeks significantly improved survival, with 80% of tumours eradicated after 28 days [61]. A Phase1 clinical trial is currently investigating GD2-CAR-T cells for GD2-positive pBTs (NCT04099797). Patients will receive chemotherapy drugs, including Cyclophosphamide and Fludarabine, before IV infusions of GD2-C7R T cells. In particular, GD2-C7R T cells are produced by infecting GD2-CAR-T cells with a retroviral vector carrying the *C7R* gene, aiming to improve their survival and tumour-killing capacity. However, one challenge of CAR-T cell therapy is severe side effects such as cytokine release syndrome (CRS), which has been reported in patients with chronic lymphocytic leukaemia [62]. Currently, iC9-GD2-CAR-T cells, which include an inducible Caspase 9 gene, are under investigation in paediatric and young adults with relapsed or refractory malignant CNS tumours (NCT05298995). Caspase 9 expression allows for controlled induction of apoptosis in CAR-T cells, acting as a safety mechanism to prevent toxicity such as CRS. Additionally, GD2-CAR-T cells are also being studied for H3K27M-mutant DIPG and DMG tumours in children and adults (NCT04196413). In this study, autologous T cells are transduced with a 142a-CD8-BBZ-iCasp9 retroviral vector, permitting controlled elimination of GD2-CAR-T cells to prevent toxicity. Additionally, another important limitation observed in patients receiving CAR-T cell treatment is antigen escape, which occurs when tumour cells lose or downregulate the expression of target antigens [56]. For example, one study reported that recurrent GBM cells exhibited reduced expression of IL13Ra2 following treatment with IL13Ra2-specific CAR-T cells [63]. Hence, this highlights a critical challenge that must be considered when using CAR-T cells for the potential treatment of pBTs.

### 4.3. Oncolytic Viruses

Oncolytic viruses are an additional form of immunotherapy, which could be used for the treatment of pBTs. These viral agents are genetically modified to infect and replicate inside tumour cells, leading to the lysis of tumour cells without affecting healthy cells [64]. T-VEC, an oncolytic herpes simplex virus, is FDA-approved for metastatic melanoma and has demonstrated good tolerability with few side effects, together with an improved durable response rate and overall survival [65].

Pelareorep, an oncolytic reovirus, preferentially replicates in *ras* mutant cells to induce an anti-tumour response. In a study of 15 patients with newly diagnosed glioblastoma, pelareorep given with GM-CSF (granulocyte-macrophage colony-stimulating factor) and chemoradiotherapy was well tolerated [66]. In another trial, pelareorep plus GM-CSF was tested in six patients with high-grade pBTs (NCT02444546). The therapy was also well tolerated, supporting further development as a future treatment for pBTs [67].

Other novel oncolytic viruses are being explored, including the Ad-TD-nsIL12 virus tested for safety and toxicity in patients with progressive DIPG (NCT05717699).

G207, an oncolytic herpes simplex virus-1 (HSV), selectively infects and lyses tumour cells to stimulate an anti-tumour immune response. A Phase1 clinical trial of G207 with radiation in 12 patients with HGG (aged 7–18 years old) showed good tolerability, no serious adverse events, and a median overall survival of 12.2 months compared with 5.6 months. Importantly, tumour-infiltrating lymphocytes were significantly increased after treatment [68]. Combination therapy of G207 with a single low dose of radiation is now being studied in paediatric patients with progressive cerebellar brain tumours (NCT03911388) and recurrent pHGG (NCT04482933).

## 5. Enhancing Efficacy Through Combination Therapy

**Figure 6:**
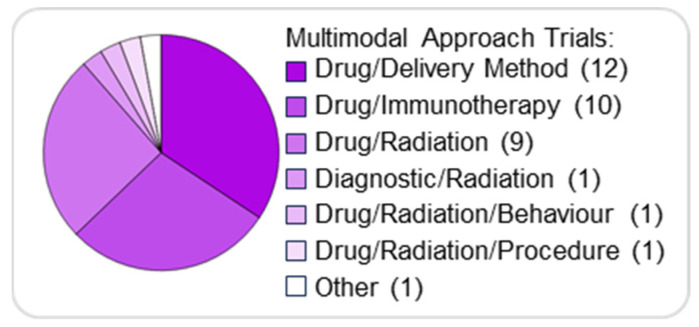
Combination therapies in paediatric brain tumours. Pie chart illustrating the distribution of combination approaches for treating pBTs.

Therapeutic approaches involving the combination of drugs are increasingly recognised as an effective cancer treatment as they can target multiple pathways essential for tumour proliferation and growth. This approach aims at improving treatment efficacy and obtaining a cooperative effect while overcoming or reducing drug resistance that frequently develops with monotherapies [69]. Various combination strategies are being explored as well as utilised in pBT treatment. As an example, the chemotherapy agent Vincristine has been used in combination with the Hedgehog pathway inhibitor Vismodegib [70]. Targeted therapies have also been combined for pBTs, as seen in the combination of dabrafenib with trametinib, BRAF and MEK inhibitors (respectively) for paediatric patients with low-grade glioma [71]. Among the analysed clinical trials, we found that combination treatments imply mainly: (i) conventional chemotherapy agents combined with targeted therapy drugs and (ii) a combination of multiple targeted therapy drugs (Figure 6). Chemotherapeutic agents are combined with inhibitors of several targets discussed previously, including kinases (e.g., CDK4/6, mTOR, BRAF) and HDAC, while the combination of targeted therapies frequently includes mTOR inhibitors.

### Multimodal Combination Approaches: Delivery, Radiation, and Immunotherapy

In addition to pharmacological combinations, several multimodal strategies are being explored to enhance drug penetration, reduce toxicity, and improve overall treatment outcomes. Besides the combination of different drugs, we also found several combinations of treatment options in which a patient is given two or more different interventions.

The most common type of combination therapy involves using a drug and a delivery method (12/35, 34%), followed by a drug and immunotherapy (10/35, 29%), and a drug and radiation therapy (9/35, 26%) (Figure 6).

The BBB prevents many drugs from reaching the brain; implementing drug delivery systems is an important aspect of BT treatment. This approach involves combining therapies, such as drugs or immunotherapies, with novel delivery methods to enhance their efficacy [72]. One such method is convection-enhanced delivery, which precisely infuses drugs into the brain parenchyma. In a pre-clinical study, the combination of MTX110, an aqueous formulation of the HDACi Panobinostat, with convection-enhanced delivery showed therapeutic efficacy and reduced toxicity in primary paediatric DIPG cell lines [73]. This combination therapy was further assessed in clinical trials with some modifications, with two studies showing safety and tolerability [74,75].

Radiation therapy, despite being renowned for its neurotoxic effects, remains a significant part of pBT treatment. In certain malignancies, such as DMG, it is the only treatment that increases survival. The mechanism of action of radiation therapy involves the delivery of ionising radiation that induces DNA damage in both healthy and malignant cells. Extensive studies have investigated the complex interactions among radiation therapy, the immune system, the TME, and the DNA damage response elicited by radiation and chemotherapeutic agents. The interplay between these factors suggests that combining radiation therapy with other treatment approaches improves efficacy and outcomes [76]. Similarly, combining drugs with immunotherapies targets different mechanisms and pathways while stimulating the immune system, enhancing tumour cell death.

Combination therapies can increase treatment efficacy in pBTs by targeting multiple oncogenic pathways. However, combining treatments, especially those with known adverse effects such as chemotherapy and radiation, may increase toxicity and adverse effects. It will be fundamental to evaluate the safety of these therapies both alone and in combination within the paediatric population.

## 6. Diagnostic and Personalised Interventions for Paediatric Brain Tumours

The therapeutic approaches that we have described are closely linked to advances in diagnostic imaging and molecular profiling, which are paving the way for precision medicine in pBTs. Diagnostic interventions made up 34/252 (13%) of the clinical trials considered.

### 6.1. Imaging

The main diagnostic intervention was imaging agents (23/34, 68%), which can be used in imaging studies such as PET, CT, and MRI. The most common imaging agents were tracers used for PET imaging (12/23, 52%). PET imaging provides information about the biochemical and metabolic activity of tissues. This can be useful for BTs, as it enables earlier detection of the metabolic changes that tend to precede structural ones. PET imaging involves the use of radiotracers, which label cells and emit positrons that are detected by the PET scanner, producing images that show functional brain activity. The diagnostic gold standard for imaging a suspected BT is MRI, which provides information on brain structures and can depict features of a BT, such as location and size [77]. Contrast-enhanced MRI is often used to visualise BTs, particularly to differentiate BTs from other brain tissue [78].

PET radiotracers can cross the BBB and/or target mechanisms that are unaffected by an intact BBB and not accessible by MRI contrast agents [79]. Studies comparing or correlating PET and MRI consistently report that the two modalities offer distinct but complementary insights [80]. Consequently, hybrid PET/MRI has been recognised as an ideal diagnostic approach for imaging pBTs, combining PET’s functional information with MRI’s anatomical detail to improve diagnosis and management [81].

We found that imaging agents accounted for most of the diagnostic interventions, highlighting the important role of imaging in the diagnosis and monitoring of pBTs. However, it is important to consider both the benefits and risks of these methods in paediatric neuro-oncology, where the patient population is often vulnerable. Paediatric patients are often sedated during scans, like MRI, to ensure they remain still and to shorten scan time. Adverse events from sedation, though uncommon, typically include airway and respiratory events, such as wheezing [82]. To mitigate these risks, alternative strategies, for example, the use of a mock scanner before MRI, have been explored and found to be beneficial. This approach aims to help familiarise patients with the scanning environment and reduce stress, allowing imaging to be conducted without sedation or with minimal sedative doses [83]. Furthermore, there are risks associated with imaging techniques themselves. Use of contrast agents, for instance, carries the risk of allergies, nephrogenic systemic fibrosis, and gadolinium deposition [84]. These findings emphasise the need to continue researching the safety of imaging agents, especially given their essential role in the diagnosis and management of paediatric BTs.

### 6.2. Molecular Profiling and Precision Medicine

Personalised interventions accounted for 16/252 (6%) of the clinical trials, of which the main type was molecular profiling (15/16, 94%). The field of oncology has recently shifted towards precision medicine, a treatment approach which emphasises tailoring treatments based on a patient’s specific molecular and/or cellular features. Molecular profiling can identify these individual markers, allowing clinicians to personalise cancer treatment and management [85]. pBTs display molecular profiles that are distinct from those of adult BTs, underscoring the need for precision medicine. Targeted therapeutic strategies tailored to the unique molecular profiles of pBTs have the potential to enhance treatment efficacy while reducing both short- and long-term adverse effects [86]. A notable example is PNOC003, a precision medicine trial in recently diagnosed DIPG, where RNA and whole-exome sequencing were performed on a pre-treatment tumour biopsy to develop a tumour molecular profile. This was then used to guide treatment as recommended by the specialised board [87]. Although personalised treatments in pBTs only represent a small proportion of clinic trials, their presence is growing, demonstrating a shift towards precision medicine.

Despite the exciting potential of personalised therapies, there are challenges to their use. Analysing tumour molecular profiles as well as developing individualised treatment may require time, as well as delay the initiation of therapy. This is particularly concerning for aggressive pBTs, where commencing treatment early can be essential to prevent progression of the malignancy. Furthermore, personalised therapies can be expensive and require many resources. Lastly, the molecular targets identified through analysis may not have approved or effective therapies, which limits the applicability.

### 6.3. Other Intervention Options Available for Paediatric Brain Tumours

The other interventions each accounted for less than 5% of all clinical trials. Standalone radiation therapy represents 8/256 (3%) of all interventions, while devices accounted for 7/256 (3%) and procedures for 6/256 (2%). Dietary interventions were the least common as 4/256 (2%) of the clinical trials. Notably, all four dietary interventions involved ketogenic diets. The ketogenic diet is a low-carbohydrate, high-fat diet recognised as a pharmaconutritional therapy [88]. Ketogenic diets have been explored as an adjuvant therapy for brain tumours, as they can influence the metabolic and epigenetic changes characteristic of cancer [89,90].

## 7. Conclusions

Over the past decade, the range of treatment options being trialled for pBTs has diversified, with new therapeutic avenues that could complement conventional surgery, chemotherapy, and radiotherapy. Nevertheless, most interventions remain in the early phases of clinical evaluation, reflecting the unique challenges of the paediatric neuro-oncology field, including the small patient population, tumour heterogeneity, and the regulatory and ethical complexities of developing drugs for children. Kinase inhibitors targeting CDK4/6, mTOR, and MAPK/ERK pathways are the most frequently explored in clinical trials, highlighting their relevance to pBT biology. However, challenges such as BBB penetrance, mechanisms of resistance, and long-term toxicity will require careful consideration as these therapies progress. Additionally, several epigenetic modulators, particularly HDAC inhibitors, are currently under investigation, reinforcing the fundamental role of epigenetic mechanisms in pBT formation and maintenance. Immunotherapies, including CAR-T cells, oncolytic viruses, and cancer vaccines, represent another promising therapeutic avenue, provided that these approaches can overcome the immunosuppressive microenvironment and the heterogeneity in antigen expression observed in pBTs. Finally, advances in diagnostics, particularly PET/MRI and molecular profiling, will enable more precise patient stratification and support adaptive therapeutic strategies. Integration of molecular insights with rationally designed single or combination therapies, alongside advanced drug delivery approaches and international trial networks, will be critical to ensure progress in the treatment of pBTs and to improve both survival and quality of life.

## Data Availability

No new data were created or analyzed in this study.

## Supplementary Materials

The following supporting information can be downloaded at: https://www.mdpi.com/article/10.3390/pharmaceutics17121561/s1.
